# Meningitis registry of hospitalized cases in children: epidemiological patterns of acute bacterial meningitis throughout a 32-year period

**DOI:** 10.1186/1471-2334-7-101

**Published:** 2007-08-30

**Authors:** Maria N Theodoridou, Vasiliki A Vasilopoulou, Erato E Atsali, Anastasia M Pangalis, Glyceria J Mostrou, Vassiliki P Syriopoulou, Christos S Hadjichristodoulou

**Affiliations:** 1 First Department of Pediatrics, Aghia Sofia Children's Hospital, University of Athens 11527, Greece; 2 Microbiology Laboratory, Aghia Sophia Children's Hospital, Athens 11527, Greece; 3 Department of Hygiene and Epidemiology, University of Thessaly, Larissa 41222, Greece

## Abstract

**Background:**

Bacterial meningitis remains a source of substantial morbidity and mortality in childhood. During the last decades gradual changes have been observed in the epidemiology of bacterial meningitis, related to the introduction of new polysaccharide and conjugate vaccines. The study presents an overview of the epidemiological patterns of acute bacterial meningitis in a tertiary children 's hospital during a 32-year period, using information from a disease registry. Moreover, it discusses the contribution of communicable disease registries in the study of acute infectious diseases.

**Methods:**

In the early 1970s a Meningitis Registry (MR) was created for patients admitted with meningitis in Aghia Sofia Children's Hospital in Athens. The MR includes demographic, clinical and laboratory data as well as treatment, complications and outcome of the patients. In 2000 a database was created and the collected data were entered, analyzed and presented in three chronological periods: A (1974–1984), B (1985–1994) and C (1995–2005).

**Results:**

Of the 2,477 cases of bacterial meningitis registered in total, 1,146 cases (46.3%) were classified as "probable" and 1,331 (53.7%) as "confirmed" bacterial meningitis. The estimated mean annual Incidence Rate (IR) was 16.9/100,000 for bacterial meningitis, 8.9/100,000 for *Neisseria meningitidis*, 1.3/100,000 for *Streptococcus pneumoniae*, 2.5/100,000 for *Haemophilus influenzae *type b (Hib) before vaccination and 0.4/100,000 for Hib after vaccination. *Neisseria meningitis *constituted the leading cause of childhood bacterial meningitis for all periods and in all age groups. Hib was the second most common cause of bacterial meningitis before the introduction of Hib conjugate vaccine, in periods A and B. The incidence of bacterial meningitis due to *Streptococcus pneumoniae *was stable. The long-term epidemiological pattern of *Neisseria meningitidis *appears in cycles of approximately 10 years, confirmed by a significant rise of IR in period C. The Case Fatality Rate (CFR) from all causes was 3.8%, while higher CFR were estimated for *Streptococcus pneumoniae *(7.5%, RR=2.1, 95% CI 1.2–3.7) and *Neisseria meningitidis *(4.8%, RR=1.7, 95% CI 1.1–2.5) compared to other pathogens. Moreover, overall CFR varied significantly among the three time periods (p = 0.0015), and was estimated to be higher in period C.

**Conclusion:**

By using the MR we were able to delineate long-term changes in the epidemiology of bacterial meningitis. Thus the MR proved to be a useful tool in the study and the prevention of communicable diseases in correlation with prevention strategies, such as vaccinations.

## Background

In the pre-antibiotic era, bacterial meningitis was a fatal disease. In our days, despite increased availability of potent antimicrobials and sophisticated intensive care units, bacterial meningitis continues to be a significant cause of childhood morbidity and mortality [1,2]. This is reflected in the fact that meningitis continues to be present in the ten leading causes of death in children of high-income countries [3].

Many clinical and etiologic studies performed over the past 30 years have demonstrated that *Haemophilus influenzae *type b (Hib), *Neisseria meningitidis *and *Streptococcus pneumoniae *were the most common causative organisms of bacterial meningitis worldwide [1,2,4]. The development and implementation of a conjugate vaccine against Hib in the early 1990's have contributed directly to changes in the epidemiological profile of meningitis caused by Hib, in the countries that adopted universal immunization [4-6]. More recently, serogroup C meningococcal conjugate vaccine and pneumococcal heptavalent conjugate vaccine were licensed and are expected to influence the epidemiology of the disease. Therefore, a review of the epidemiology of bacterial meningitis is important in order to make rational decisions concerning future prevention and control strategies.

In Greece, studies on the epidemiology of childhood bacterial meningitis have largely focused on single causative organisms and specific geographic areas, while a nationwide surveillance system was not established before 1993 – when the National Meningococcal Reference Laboratory became operative.

Medical registries are defined as records of all new cases of selected diseases in defined populations that are prospectively monitored. They have been extensively used in the research of chronic diseases, while also communicable disease registries are being increasingly used. In the early 1970s, the creation of a Meningitis Registry (MR) in the Infectious Diseases Department of Aghia Sofia Children's Hospital in Athens, responded to the need for comprehensive epidemiological information on childhood meningitis in Greece. This report presents the 32-year MR findings regarding the epidemiological features of bacterial meningitis.

## Methods

### Study setting

Aghia Sofia Children's Hospital is a 250-bed pediatric university teaching hospital, which serves as a major referral center. The hospital has an average of 20,000 admissions annually, 4% of them treated in the Infectious Diseases Department. It is important to mention that Athens metropolitan pediatric population is covered by two more pediatric hospitals and few pediatric clinics located in general hospitals.

Nevertheless, the majority of meningitis cases occurring in Athens metropolitan area, as well as those referred from central and south Greece and most of the Aegean Sea islands of the country were transferred and treated in the Infectious Diseases Department of Aghia Sofia Children's Hospital. During the last decade this pattern has changed, leading to a more wide distribution of meningitis cases at different facilities and a subsequently increasing number of meningitis cases treated in the other pediatric clinics. Taking into account the above information, it is postulated that, concerning specifically meningitis cases, the hospital served approximately 100% of the pediatric population of metropolitan Athens in period A, approximately 65% in period B and approximately 55% in period C.

### Data collection

In the early 1970s, a MR was created for patients admitted with meningitis in the Infectious Diseases Department of Aghia Sofia Children's Hospital. In the Registry Form (RF) around 50 parameters were included: demographic data (full name, sex, date of birth, father occupation, home address, telephone number, date of entry and date of discharge) and clinical data (preadmission antibiotic therapy, comorbid conditions, fever, headache, vomiting, meningeal signs, petecchiae, seizures, bulging fontanelle, grumbling, poor feeding, collapses or coma – on the day of admission, as well as for the following days). Moreover MR included laboratory results from Cerebrospinal Fluid (CSF) examination (white cell count, polymorphonuclear and lymphocyte count, glucose, protein, Gram stain, culture, latex, Polymerase Chain Reaction [PCR]), blood examination (hemoglobin, haematocrit, white cell count, polymorphonuclear and lymphocyte count, culture, PCR, prothrombin time), urine analysis, nasopharyngeal culture, stool culture and Mantoux. Finally, type and duration of provided treatment, discharge diagnosis, outcome and sequelae were recorded.

For each case the attending physician completed the RF upon discharge of the patient. Follow up visits were held up to 3 months after discharge and all additional information was recorded. To attain completeness, RFs were also created for two categories of patients who died from bacterial meningitis in the hospital without being treated in the Infectious Diseases Department. The Intensive Care Unit (ICU) records were reviewed to identify patients who were admitted directly to the ICU and died there from bacterial meningitis. Secondly, patients who died in the emergency department or were dead on admission with signs of bacterial meningitis or meningococcal septicemia were also recorded, after the review of hospital death records. Despite those cases were not hospitalized, a special RF was created and was included in the MR.

In 2000 a computerized database was created using Epi-nfo epidemiological package (version 3.3.2 – CDC – Atlanta) and for one year the already compiled RFs were entered into the database. Moreover, RFs were entered prospectively for the years 2000–2005.

### Case definition – Inclusion criteria

A "case" was defined as any patient treated in the Infectious Diseases Department of Aghia Sofia Children's Hospital from January 1^st ^1974 until December 31^st ^2005, who was aged one-month-old to fourteen-years-old and was diagnosed with bacterial meningitis.

"Probable" bacterial meningitis cases were defined -according to World Health Organization (WHO) criteria- those presenting clinical symptoms of meningitis (i.e. fever, headache, stiff neck, bulging fontanel or altered mental status) and CSF with an elevated protein (> 100 mg/dl), decreased glucose (< 40 mg/dl) or leukocytosis (> 100 WBC/mm^3^) with at least 80% neutrophils and lacking an identifiable bacterial pathogen [7].

"Confirmed" bacterial meningitis cases were defined according to WHO case definition criteria [7]: children presenting with clinical symptoms of meningitis (i.e. fever, headache, stiff neck, bulging fontanelle or mental status changes) and identification of bacteria *directly *(by culture or PCR from blood or CSF, or by culture from the petecchial lesions), or *indirectly *(by latex test, countercurrent immunoelectrophoresis, Fadebact, or Gram stain smear of blood or CSF). The "Confirmed" bacterial meningitis cases were further divided into the following groups, representing the most commonly isolated pathogens: *Neisseria meningitidis, Streptococcus pneumoniae, Haemophilus influenzae *type b and a fourth group "confirmed bacterial meningitis due to other bacteria" including all other isolated pathogens.

"Probable" meningococcal meningitis cases were defined -according to WHO case definition- those with clinical findings (fever > 38°C, hemorrhagic rash, circulatory collapse or coma) and laboratory findings compatible with meningococcal meningitis disease (CSF protein > 100 mg/dl, CSF glucose < 40 mg/dl or > 100 CSF WBC/mm^3 ^with at least 80% neutrophils), although *Neisseria meningitidis *had not been isolated [8].

Deaths were attributed to bacterial meningitis if the patient died within one month of receiving in hospital antimicrobial therapy in hospital and if bacterial meningitis was the direct or predisposing cause of death.

### Exclusion criteria

All patients that had been diagnosed repeatedly with events of bacterial meningitis due to structural defects of the central nervous system were excluded from the study. Meningitis cases caused by *Mycobacterium tuberculosis *were also excluded, as well as cases of patients younger than 1 month old.

### Study periods

In order to facilitate data analysis and taking into consideration the fact that the conjugate Hib vaccine was introduced in Greece in mid 1994, cases were divided by date of admission, in three time periods (of 11, 10 and 11 years respectively): period A (1974 – 1984), period B (1985 – 1994) and period C (1995–2005). Periods A and B correspond to the time before the implementation of conjugate Hib vaccine, while period C corresponds to the time when immunization for Hib was practiced routinely in Greece.

### Data analysis

Statistical analyses were performed with Epi-nfo epidemiological package (version 3.3.2 – CDC – Atlanta). The chi-square test and the Fischer exact test were used to compare qualitative variables. All results for continuous variables are expressed as means (Standard Deviation, SD). The student *t*-test, or Mann-Whitney test were used for quantitative variables. The level of 0.05 was used for statistical significance. Risk ratio (RR) with 95% confidence intervals (95% CI) were also calculated. Annual incidence was calculated per 100,000 children less than 14 year of age served by Aghia Sofia Children's Hospital. Population figures were extrapolated based on 1971, 1981, 1991 and 2001 census [9-12]. Potential years of life lost were calculated using the WHO life expectancy at birth for males and females in Greece [13].

## Results

During the 32 years of the MR a total of 3,495 cases were recorded, 2,477 (70.87%) of which met the criteria for bacterial meningitis. "Probable bacterial meningitis cases" were considered 1,146 (46.3%), while "confirmed bacterial meningitis cases" were considered 1,331 (53.7%). A single bacterial organism was identified in all "confirmed bacterial meningitis cases". The organism was identified directly in 1,073/1,331 cases (80.6%) out of which 789/1,073 cases (73.5%) were found positive only in CSF culture, 183/1,073 (17.1%) were found positive both in CSF and blood culture, whereas the remaining 101/1,073 (9.4%) were positive only in blood culture. A total of 258/1,331 cases (19.4%) were identified indirectly.

**Figure 1:**
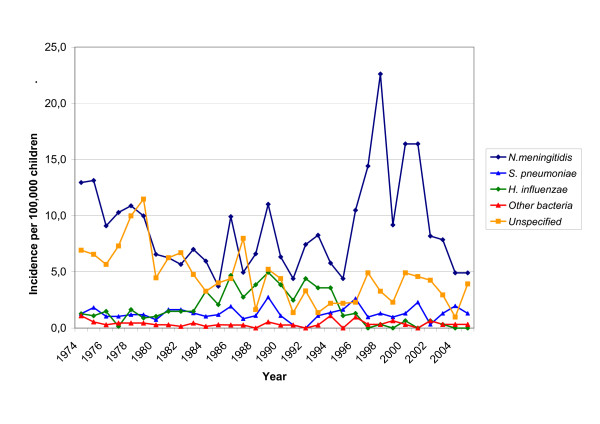
IR of bacterial meningitis according to etiology from 1974 to 2005.

The agents most commonly associated with bacterial meningitis were *Neisseria meningitidis *(Incidence Rate [IR] 8.9 per 100,000 children), Hib (IR 1.7 per 100,000 children) and *Streptococcus pneumoniae *(IR 1.3 per 100,000 children), while in 30% of cases no bacterial pathogen was identified. Total number and mean annual IR of bacterial meningitis cases in the three periods of the study are presented in Table 1. Moreover, the yearly distribution of IR of bacterial meningitis according to pathogenic agent is shown in Figure 1.

**Table 1:** Bacterial meningitis in three periods according to pathogen

**Organism**	**1974–1984**			**1985–1994**			**1995–2005**			**Total**		
	
	**N**	**%**	IR*	**N**	**%**	IR*	**N**	**%**	IR*	**N**	**%**	IR*
***N. meningitidis*- probable**	238	18.0	**3.4**	63	10.5	**1.6**	113	20.3	**3.3**	414	16.7	**2.8**
***N. meningitidis*- confirmed**	386	29.3	**5.5**	197	32.8	**5.2**	255	45.7	**7.6**	838	33.8	**6.1**
***N. meningitidis*- total**	624	47.3	**8.9**	260	43.3	**6.8**	368	65.9	**10.9**	1252	50.5	**8.9**
***S. pneumoniae***	90	6.8	**1.3**	46	7.7	**1.2**	50	9.0	**1.5**	186	7.5	**1.3**
***H. influenzae *type b**	100	7.6	**1.4**	138	23.0	**3.6**	14	2.5	**0.4**	252	10.2	**1.7**
Other bacteria***	29	2.2	**0.4**	13	2.2	**0.3**	13	2.3	**0.4**	55	2.2	**0.4**
Unspecified**	476	36.1	**6.7**	143	23.8	**3.6**	113	20.3	**3.3**	732	29.6	**4.6**
**Total**	1319	100	**18.7**	600	100	**15.5**	558	100	**16.5**	2477	100	**16.9**

* Mean annual Incidence Rate per 100,000 children

** "Probable" bacterial meningitis cases not including "probable" meningococcal meningitis cases

*** group B *Streptococcus, Salmonella *spp, *Streptococcus *spp, *Pseudomonas aeruginosa, Escherichia coli, Staphylococcus *spp, *Brucella melitensis, Klebsiella pneumoniae, Acinetobacter anitratus, Enterobacter cloaca, Mycoplasma pneumoniae, Proteus *spp, *Rickettsiae *spp

Males (58.7%) outnumbered females overall, while the mean annual IR of bacterial meningitis (per 100,000 children) was estimated 19.1 for males and 14.6 for females. Age specific rates for bacterial meningitis varied with pathogen (Table 2). The mean age was 3.1 years (SD 3.6; median 2; range 1 month to 14 years). Younger than 5 years were 71.4% of patients (1,718 out of the 2,406 children whose age was available). Patients aged < 1 year accounted for one third (786 – 32.7%), while patients 10 years and older accounted for 8.6% of the series.

**Table 2:** Meningitis cases in the three most common pathogens according to age group, in three periods

**Organism**		N. Meningitidis*			***S. Pneumoniae***			***H. Influenzae***		
**Age Group**	**Period**	**N**	**%**	IR**	**N**	**%**	IR**	**N**	**%**	IR**

**1 to 11 **	1974–1984	202	63.3	32.8	45	60.8	7.3	41	40.2	6.5
**months**	1985–1994	69	21.6	27.9	15	20.3	5.6	55	53.9	22.9
	1995–2005	48	15.0	18.9	14	18.9	5.5	6	5.9	2.3
**1 to 4 **	1974–1984	274	50.3	14.4	26	38.8	1.3	49	38.6	2.6
**years**	1985–1994	136	25.0	16.7	14	20.9	1.7	72	56.7	8.7
	1995–2005	135	24.8	16.3	27	40.3	3.3	6	4.7	0.7
**5 to 9 **	1974–1984	93	38.4	4.1	10	40.0	0.4	7	43.8	0.3
**years**	1985–1994	38	15.7	3.0	8	32.0	0.6	8	50.0	0.6
	1995–2005	111	45.9	10.1	7	28.0	0.6	1	6.3	0.1
**10 to 14 **	1974–1984	36	33.0	1.6	7	46.7	0.3	1	50.0	0.0
**years**	1985–1994	13	11.9	0.9	7	46.7	0.5	1	50.0	0.0
	1995–2005	60	55.0	5.1	1	6.7	0.1	0	0.0	0.0
**Total**	1974–2005	1252	100.0	8.9	186	100.0	1.3	252	100.0	1.7

* *N. Meningitidis *includes probable and confirmed meningococcal meningitis cases

** Mean annual Incidence Rate per 100,000 children

During the 32 years of the study, 95 patients died in total. The Case Fatality Rate (CFR) of bacterial meningitis from all causes was estimated 3.8% (95% CI 3.1–4.7) The CFR varied significantly according to organism (p = 0.0001) and among the three time periods (p = 0.0015). The CFR was estimated higher during period C (5.4%, 95% CI 3.7–7.7) compared to periods A and B (RR 1.4, 95% CI 1.0–1.9), while CFR was estimated lower during period B (1.5%, 95% CI 0.7–2.9) compared to periods A and C (RR 0.4, 95% CI 0.2–0.7). The potential years of life lost were 7,250 in total. Potential years of life lost per period were estimated as follows: A 4,320, B 700 and C 2,230. The mean length of hospitalization was 11.7 days (SD 6.1) and decreased gradually from period A to period C 12.4, 11.8 and 9.7 days respectively (p < 0.0001). Moreover, infants under 1 year of age were hospitalized for longer (14.0 days) compared to older children (p < 0.0001). Patients who died from bacterial meningitis were hospitalized for a mean 3.8 days (SD 6.7 days), ranging from 0 to 38 days. Only one patient who died, in period A, from pneumococcal meningitis complicated with subdural effusion was hospitalized for more than one month. Table 3 presents the absolute number of fatal cases, CFR and mean duration of hospitalization.

**Table 3:** Case fatality rate and duration of hospitalization

	**Case fatalities**			**Days of hospitalization**		
	
**Organism**	**N**	**CFR% (95% CI)**	**Relative Risk (95% CI)**	**Mean (SD)**	**Range**	**N**
***N. meningitidis *– probable**	46	11.1 (8.3–14.6)	4.7 (3.2–6.9)			
***N. meningitidis*- confirmed**	14	1.7 (1.0–2.9)	0.3 (0.2–0.6)			
***N. meningitidis*- total**	60	4.8 (3.7–6.2)	1.7 (1.1–2.5)	10.5 (5.1)	0 – 65	1242
***S. pneumoniae***	14	7.5 (4.2–12.3)	2.1 (1.2–3.7)	15.8 (9.4)	0 – 95	184
***H. influenzae *type b**	2	0.8 (0.1–2.8)	0.2 (0.0–0.8)	14.5 (5.1)	1 – 47	252
**Other bacteria**	4	7.3 (2.0–17.6)	1.9 (0.7–5.1)	20.3 (12.4)	0 – 68	53
**Unspecified**	15	2.0 (1.2–3.4)	0.4 (0.3–0.8)	11.2 (5.1)	0 – 48	725
**Total**	95	3.8 (3.1–4.7)		11.7 (6.1)	0 – 95	2456

**Figure 2:**
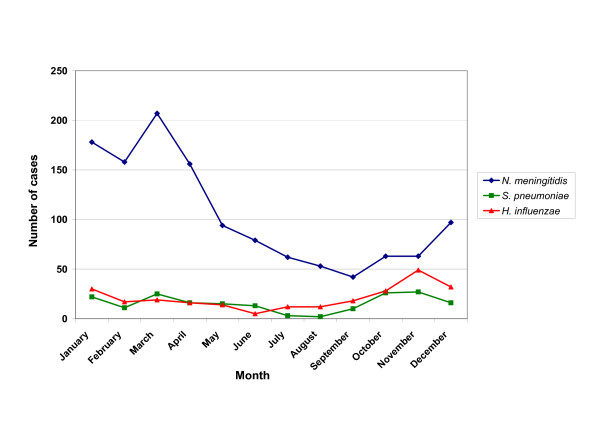
Monthly variation of the three most common pathogens of bacterial meningitis.

All pathogens showed some seasonal variation. Sixty-nine percent (69.0%) of the cases were diagnosed between October and April. Monthly distribution of *Neisseria meningitidis*, *Streptococcus pneumoniae *and Hib is presented in Figure 2.

### *Neisseria meningitidis*

Meningococci constituted the leading cause of bacterial meningitis cases treated in the Infectious Diseases Department of Aghia Sofia Children's Hospital, during the last three decades and in all age groups. More than half of bacterial meningitis cases registered were attributed to *Neisseria meningitidis *(1,252 out of 2,477 – 50.5%). The mean annual IR of total meningococcal meningitis cases was estimated 8.9 per 100,000 children. In the third period a 38.0% increase of the mean annual IR was estimated (from 7.9 in periods A and B to 10.9 per 100,000 children in period C). This rise was more pronounced in patients with confirmed meningococcal meningitis (Table 1).

**Figure 3:**
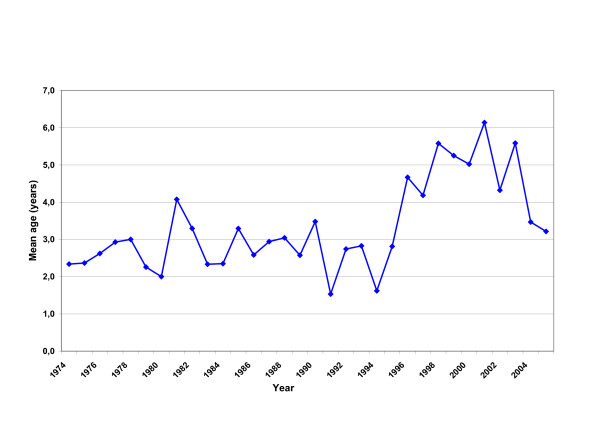
Mean age of meningococcal cases from 1974 to 2005.

Most cases due to *Neisseria meningitidis *occurred in infants and young children: 26.3% occurred in infants < 1 year and 71.1% in children < 5 years of age. Infants < 1 year of age had the highest age specific IR (32.8, 27.9 and 18.9 per 100,000 children in periods A, B and C). The mean age of *Neisseria meningitidis *cases, approximately 2.7 years (SD 3.2) in periods A and B, marked a significant increase in period C up to 4.9 years (SD 3.9), as presented in Figure 3 (p < 0.0001). This observation applies, to "confirmed" and "probable" meningococcal disease as well. In total, males (55.1%) outnumbered females.

The overall CFR for *Neisseria meningitidis *was estimated 4.8% (95% CI 3.7–6.2), while it was estimated 1.7% (95% CI 1.0–2.9) for "confirmed" meningococcal meningitis. The higher CFR (11.1%, 95% CI 8.3–14.6) was observed in the "probable" *Neisseria meningitidis *cases compared to all other bacterial meningitis cases (RR 4.7, 95% CI 3.2–6.9). Interestingly, higher rates of case fatality were estimated in period C (6.3%, 95% CI 4.1–9.4), compared to 5.3% (95% CI 3.7–7.4) and 1.5% (95% CI 0.4–3.9) in periods A and B. Age specific CFR was higher in the 1–4 years (7.4%, 95% CI 3.6–13.2) and 10–14 years (10.0%, 95% CI 3.8–20.5) age groups.

Of the 838 confirmed meningococcal meningitis cases serogroup was available for only 321 (38.3%), with the predominant isolates being B (58.6%), C (23.4%) and A (10.9%). Serogroup differed among the three periods of the study. In period A 75 cases (70.8%) of serogroup B, 16 cases (15.1%) of serogroup A and 13 cases (12.3%) of serogroup C were isolated. Correspondingly, in period B 34 cases (49.3%) of serogroup B, 32 cases (46.4%) of serogroup C and 1 case (1.4%) of serogroup A were isolated. Finally, in period C 79 cases (54.1%) of serogroup B, 30 cases (20.5%) of serogroup C and 18 cases (12.3%) of serogroup A were isolated.

Meningococcal meningitis cases demanded the shortest duration of hospitalization (10.5 ± 5.1 days) among all other causes (Table 3). A seasonal pattern of meningococcal meningitis was observed, with a peak during winter and spring, presenting a maximum from January to March (Figure 2).

### *Streptococcus pneumoniae*

Pneumococcal meningitis occurred in 186 cases out of 2,477. The mean annual IR in total was 1.3 per 100,000 children. The incidence of pneumococcal meningitis remained relatively stable during the three periods of the study. Overall, the risk of meningitis from *Streptococcus pneumoniae *was higher in the < 1 year and < 5 years age groups. The mean annual IR observed in infants < 1 year of age was 7.3, 5.6 and 5.5 per 100,000 in the three periods. Respectively, IR in children < 5 years of age was estimated 2.8, 1.8 and 2.1 per 100,000 children for periods A, B and C.

*Streptococcus pneumoniae *meningitis affected children with a mean age of 2.7 years (SD 3.6), which varied from 2.2, 3.7 and 2.4 in periods A, B and C respectively. Two thirds of pneumococcal meningitis cases were males (male to female ratio 2.1:1).

The overall CFR was 7.5% (95% CI 4.2–12.3), which was the highest among the three most common pathogens (RR 2.1, 95% CI 1.2–3.7). Interestingly, no casualty was recorded from pneumococcal meningitis during period B, while the CFR increased significantly during period C (CFR in period A 7.8%, period B 0%, period C 14.0%).

The duration of hospitalization (including casualties) was 15.8 ± 9.4 days, the second longer following "other bacterial organisms" (Table 3).

The majority of cases (127 out of 186, 68.3%) manifested between October and March, presenting with a bimodal pattern. One peak was noticed during autumn, in October and November, and a second occurred during spring, in March (Figure 2).

### *Haemophilus influenzae *type b

*Haemophilus influenzae *type b was responsible for 252 meningitis cases, constituting the second most common etiologic agent, in the fist two periods of the study. The IR of Hib meningitis began a noticeable decline after 1994, the year that the Hib conjugate vaccine was introduced. The mean annual IR for Hib before the vaccination was estimated 2.5 per 100,000 children: rather low in period A (1.4 per 100,000 children) and higher in period B (3.6 per 100,000 children). A fall up to 0.4 per 100,000 children was observed during period C. In particular, in the children < 5 years of age the mean annual IR was estimated 3.5 per 100,000 children in period A, 8.0 in period B and 0.6 in period C, presenting with an estimated 92.5% decline from period B to C.

The mean age of children affected by Hib meningitis was 1.3 years (SD 1.9), quite lower compared to the age of children affected by *Neisseria meningitidis *and *Streptococcus pneumoniae*. The 41.3% and 51.4% of children affected by Hib meningitis were aged < 1 year and 1–4 years respectively. Males (57%) were more numerous than females.

The CFR of Hib (0.8%, 95% CI 0.1–2.8) was found lower than the CFR of other pathogens (RR 0.2, 95% CI 0.0–0.8). All deaths from Hib occurred during the first period (CFR in period A 2.0%, 95% CI 0.2–7.0) and concerned children with a mean age of 8 months (SD 2.8 months, range 6–10 months). The estimated CFR in the children < 1 year and < 5 years of age was 2.0% (95%CI 0.2–6.9%) and 0.9% (95% CI 0.1–3.1) respectively.

Patients with Hib meningitis were hospitalized for a mean 14.5 ± 5.1 days, 4.0 days more than meningococcal meningitis (Table 3).

A seasonal variation in Hib meningitis cases was observed, presenting with two peaks, the first in late autumn and the second in the early spring (Figure 2).

### Bacterial meningitis due to other organisms

In total 55 cases were recorded which are being analyzed in Table 1. The overall IR of this category was 0.4 cases per 100,000 children, and was stable during all periods of the study. Group B *Streptococcus *was the most frequently isolated pathogen. Mean age of patients affected by "other bacterial organisms" was 1.3 years (SD 2.8), ranging from 1.0 month up to 11.0 years. In the four age groups (1–11 months, 1–4 years, 5–9 years and 10–14 years) the frequency of "other bacterial pathogens" was 77.4%, 7.5%, 11.3% and 3.8% respectively. Male predominance was noted (63.6%).

The overall CFR was 7.3% (95% CI 2.0–17.6), almost double compared to the average CFR estimated for bacterial meningitis (RR 1.9, 95% CI 0.7–5.1). The CFR for *Escherichia coli *was estimated 20% and the CFR for *Salmonella *spp was estimated 18.2%. All 4 deaths from bacterial meningitis due to "other bacteria" occurred during the first 2 periods of the study. Duration of hospitalization was longer (20.3 ± 12.4 days) for children with meningitis due to "other bacteria", compared to *Streptococcus pneumoniae *and Hib (15.8 ± 9.4 and 14.5 ± 5.1 days respectively) as shown in Table 3 (p < 0.0001). No seasonal pattern was observed.

### Bacterial meningitis due to unspecified pathogens

A total of 732 cases out of 2,477 (29.6%) were classified as "probable" bacterial meningitis. The overall IR of this category was 4.6 cases per 100,000 children. A significant decline was observed in the mean annual IR from period A (6.7 per 100,000 children) to period B (3.6 per 100,000 children), followed by a slight further decrease in period C (3.3 per 100,000 children).

The mean age of patients in this group was 3.7 years (SD 3.9), while the different age groups were represented as follows: < 1 year 35.2%, 1–4 years old 26.6%, 5–9 years old 26.9% and 10–14 years old 11.3%. Male to female ratio was estimated 1.7:1.

The overall CFR was estimated 2.0% (95% CI 1.2–3.4). No death in "probable" bacterial meningitis group was recorded during period C. Mean duration of hospitalization was 11.2 ± 5.1 days, ranging from 0 to 48 days.

## Discussion

This comprehensive study examined the epidemiological features of acute bacterial meningitis cases that were treated in a tertiary children's hospital from 1974 to 2005, using information prospectively collected through a MR. *Neisseria meningitidis *was determined as the main pathogen involved into the aetiology of bacterial meningitis in children throughout the 32 years of the study. Changes in the IR of bacterial meningitis, during the 3 periods that were examined, reflect the significant decline of Hib meningitis cases following the routine implementation of Hib conjugate vaccine, as well as the natural fluctuation of meningococcal meningitis. The estimated CFR of bacterial meningitis (3.8%, 95% CI 3.1–4.7) was comparable to the mean mortality rate for children reported in a meta-analysis of prospective cohorts from developed countries (4.8%, 95% CI 3.1–6.3) [14]. Other authors report CFRs up to 24% [15].

### *Neisseria meningitidis*

*Neisseria meningitidis *was found to be the leading cause of childhood bacterial meningitis in the present study during all periods and in all age groups. This finding is in line with the reports from the National Meningococcal Reference Laboratory (data for all age groups from 1997 onwards [16]) and with previous reports from specific geographic areas of the country [17-19]. In contrast to the above observation, many countries worldwide report Hib to be the most common cause of bacterial meningitis among children, in the period before the introduction of Hib vaccines [20-25]. Other countries report meningococcal meningitis to be the major cause of bacterial meningitis in young children, such as Scotland [25], Bulgaria [26] and Nigeria [27].

In common with other studies, it was found that the risk of meningococcal meningitis was higher in infants and young children aged < 5 years [25,28]. The statistically significant increase in the number of cases during period C was linked to the introduction of the new epidemic clone of *Neisseria meningitidis *C:2a:P1.2 in the years 1996–1997 [29,30], responsible for disease outbreaks also in the Czech Republic and Canada [31,32]. As already discussed, this could reflect the epidemiological pattern of meningococcal disease that appears in cycles of approximately 8–12 years [8,33]. Current findings also confirm the well-known fact that introduction of a new virulent clone in a community is followed by a shift in the age distribution towards toddlers and pre-schoolers [8,29,34]. In sporadic conditions the highest rates occur in young children. Mean age of *Neisseria meningitidis *cases increased in the current study from 2.7 (in periods A and B) to 4.9 years (in period C), as shown in Figure 3. In accordance with previous reports higher case fatality rates (6.3%) were estimated in period C, compared to 5.3% and 1.5% respectively in periods A and B [16,29]. This rise of CFR is considered as being related to the emerging new epidemic clone. A high fatal outcome from meningococcal meningitis (10%) was noticed in the 10–14 years age group during period C.

Corresponding to the findings of other reports [28,35], the remarkably high (11.1%) CFR of "probable" *Neisseria meningitidis *cases estimated represents the fulminant and acute clinical forms of the disease. In the majority of these cases clinical evolution was so rapid and devastating that antibiotic treatment was administered prior to the lumbar puncture, leading to decreased isolation rates. The introduction of more sophisticated diagnostic techniques (such as Elisa and PCR), and probably an increase in the sensitivity of classical methods (e.g. CSF and blood cultures) contributed to the reduction by 73.5% of the "probable *Neisseria meningitis*" cases from period A to period B.

According to our findings the main serogroups isolated throughout the study were A, B and C. Serogroup B was most commonly isolated during periods A and C, while during period B serogroups B and C presented with comparable rates (49.3% and 46.4%). The increase of serogroup C during 1996–7 that has been reported [16,29] is not reflected in our data, since we are analyzing cumulative data for 10 years and not analyzing data by year. Noteworthy is the considerable contribution of serogroup A in periods A and C (15.1% and 12.3%). The re-emergence of serogroup A in the late 1990s has been hypothesized to be linked to immigrants moving to Greece from Eastern European countries, where serogroup A is known to be prevalent [36].

### *Streptococcus pneumoniae*

Meningitis accounts for 10–16% of invasive pneumococcal disease [37]. In our series *Streptococcus pneumoniae *was responsible for a relatively stable proportion of bacterial meningitis cases throughout the 32 years of the study. During the last decade, following the introduction of Hib conjugate vaccine, pneumococci became the second most common etiologic agent of childhood bacterial meningitis. IR estimated for pneumococcal meningitis was slightly lower than previously reported for pediatric population in Greece [38]. The small difference between the IR in the two studies is possibly due to the fact that although they were implemented in the same hospital, a different catchment was used in order to estimate the reference population, 55% in the current study and 45% in the older study. Comparable IRs (1.6 and 1.63 per 100,000) are reported from England and Wales and Germany for children aged < 15 and < 16 years respectively [39,40]. In the present study the higher age specific IR was estimated in infants 1–11 months, ranging from 7.4, 5.6 and 5.5 per 100,000 children in periods A, B and C respectively. Similar findings from Europe report the CFR of children < 1 year ranging from 6.1 to 17.8 per 100,000 children depending on the country and the study [25,41-44].

The CFR for pneumococcal meningitis was estimated to be almost double of that of bacterial meningitis in total. Comparable CFR has been reported previously from other studies in Greece [38] and the United States [37]. Other studies mention higher CFR up to 16% for childhood pneumococcal meningitis [44]. High mortality of pneumococcal meningitis should be further investigated and to be set as a priority towards the development of improved preventive as well as therapeutic approaches.

Male to female ratio estimated in the present study exceeds by far the previously reported in Greece (2.1:1 and 1.4:1 respectively) [38]. Moreover, the mean age of children affected by pneumococcal meningitis was found higher than previously reported. The bimodal seasonal variation observed was consistent with other reports [38,43].

Although the heptavalent *Streptococcus pneumoniae *conjugate vaccine became available in the country in 2003, vaccination coverage was low during the study period. Currently presented data could prove valuable in order to compare the burden of pneumococcal meningitis in Greece before and after the implementation of conjugate pneumococcal vaccine.

### *Haemophilus influenzae *type b

The overall annual incidence for Hib meningitis in children aged 0 to 4 years in the pre-vaccination era ranged in Europe from 11 to 40 per 100,000 with an estimated average of 23 per 100,000 [5]. The reported IR of Hib meningitis in Greece in children 0 to 4 years of age before the introduction of Hib conjugate vaccine was 8.0 per 100,000 [17,45], the lowest IR observed in Europe. Several southern European and Mediterranean countries, as well as Japan, presented with comparable rates [26,46,47]. Findings from the present study during period B agree with previous reports from Greece during the pre-vaccination era [17,45]. As a result it is believed that Hib IRs during period A are underestimates. This could be attributed to improved diagnostic techniques that contributed to higher isolation and identification rates and the decline of cases of "unspecified etiology" noted in period B (Table 1).

Following the introduction of the new conjugate Hib vaccine in the late 1993 in Greece, a dramatic decline (92.5%) of Hib meningitis cases was noted in period C. Similar findings have been recorded in the United States [24], Canada [48] and several European countries [46,49,51], with reduction levels up to 98%.

During period C a total of 14 cases of Hib meningitis were recorded. Among these 14 cases during period C, 4 were recorded in 1995, 4 in 1996, 1 in 1998, 2 in 2000, 2 in 2002 and 1 in 2003, the last being the latest *Haemophilus influenzae *type b meningitis case treated in the infectious diseases department of Aghia Sofia Children's Hospital up to now. In line with other reports it was shown that more than 90% of Hib meningitis occurs in the < 5 years patient group 52–54].

The CFR estimated in the present study (0.8% in all ages and 0.9% in children < 5 years of age) was found lower compared to CFR reported from developed countries (5%) [5]. It is noteworthy that all Hib meningitis fatalities occurred in children during the first year of life, an observation that justifies the introduction of immunization early in infancy.

According to our data, emergence of other serotypes of *Haemophilus influenza *did not occur following the routine use of Hib conjugate vaccine.

### Other bacteria

Bacterial meningitis due to other bacteria affects mainly children in the first two years of life, probably reflecting the immaturity of the immune system of the host. Most authors report similar pathogens to our findings, including gram-negative and gram-positive bacteria as etiologic agents of bacterial meningitis [15,20,48]. An important observation is theoccurrence of some rare pathogens such as *Brucella melitensis*, *Mycoplasma pneumoniae *and *Rickettsiae *spp, which are uncommon causes of meningitis in childhood. Interestingly, the IR of meningitis due to other bacteria remained relatively stable during the three decades of the study, indicating that this category was not influenced by the improvement of diagnostic techniques with time.

### Bacterial meningitis due to unspecified pathogens

Approximately one out of three meningitis cases were registered as "probably" bacterial in origin, since no pathogen was isolated. The decline in the IR of meningitis cases due to unspecified bacteria, observed between period A and B, could be possibly linked to the simultaneous increase in the Hib meningitis IR, reflecting the improved isolation rates of bacterial pathogens and Hib in particular. A further reduction observed during period C is due to the introduction and routine use of methods with higher sensitivity and specificity, such as PCR, for the laboratory diagnosis of bacterial meningitis.

Following, the contribution of MR is being discussed. Medical registries are usually applied in chronic diseases such as cancer, congenital defects, primary immunodeficiencies, neurological disorders (e.g. multiple sclerosis), renal failure and coronary artery disease [55-61]. Lately, various studies concerning infectious diseases registries have been published, including chronic infections e.g. hepatitis B and HIV/AIDS [62,63], vaccine registries [64] and Epstein Barr encephalitis registry [65].

The creation of MR in order to study an acute life-threatening bacterial infection entails important benefits as well as drawbacks. For every case selected information is gathered in a systematic way and information is recorded in hard copy, in a standard RF. The collected information may lead to risk factors hypothesis generation, which can be further investigated through case control and cohort studies. Moreover, changing patterns in medical knowledge and practice are reflected (e.g. changes in definitions, diagnostic methods, and therapeutic approaches). On the other hand, the shortcomings of the MR are related with the long duration of the study and the high turnover of the hospital personnel.

This is one of the weaknesses of the present study, as it is believed to have influenced the homogeneity of the information that was gathered. Moreover, in spite of the wide variety of information that was planned to be collected, certain variables presented a high number of missing information and were excluded from the analysis.

"Another restriction of the present study is related to the changes in referral patterns of paediatric patients with bacterial meningitis in Athens metropolitan area, that leaded to a gradual decrease in the number of patients treated in our department. In order to correctly estimate the yearly Incidence Rate, it was presumed that the population served by the hospital declined gradually from 100% in period A to 65% in period B and 55% in period C, and this assumption might have affected the quality of our assessments of incidence rates. In any case the long term trend of the incidence rates is in accordance with analysis of the absolute numbers and the percentage of the total meningitis number of cases in which no population denominator is used."

## Conclusion

*Neisseria meningitidis *constituted the leading cause of bacterial meningitis in all age groups and during the whole of the 32 years studied. Variations in the incidence of meningococcal meningitis were present over time, following the well described natural cycles that characterize the epidemiology of meningococci. *Streptococcus pneumoniae *was the most lethal pathogen involved in the etiology of bacterial meningitis. *Haemophilus influenzae *type b was an important cause of morbidity before the implementation of routine vaccination with conjugate vaccine, while decreased after the vaccination introduction.

Meningitis surveillance, including the registry, is necessary to monitor the disease trend, population at risk, serotype distribution and antimicrobial susceptibility in order to implement appropriate public health interventions against invasive bacterial disease. Through the present study the MR proved to be a useful tool in the study and the prevention of communicable diseases in correlation with prevention strategies, such as vaccinations.

## Abbreviations

CFR, case fatality rate; CSF, cerebrospinal fluid; Hib, *Haemophilus influenzae *type b; ICU, intensive care unit; IR, incidence rate; MR, meningitis registry; PCR, polymerase chain reaction; RF, registry form; SD, standard deviation; WHO, World Health Organization

## Competing interests

The author(s) declare that they have no competing interests.

## Authors' contributions

MNT, VPS and CSH designed the study. MNT, VAV, EEA and GJM collected the data. AMP performed laboratory analysis. CSH and VAV performed the statistical analysis. MNT, VAV and CSH interpreted the results. MNT, VAV and CSH wrote the manuscript. VPS provided valuable insight for revising the manuscript. All authors read and approved the final manuscript.

## Pre-publication history

The pre-publication history for this paper can be accessed here:


